# Radiomics-based prediction of pituitary adenoma consistency: a systematic review and meta-analysis

**DOI:** 10.1007/s11547-026-02281-2

**Published:** 2026-09-18

**Authors:** Giancarlo Fusco, Claudio Caiazza, Renato Cuocolo, Gaetano Ungaro, Edoardo Agosti, Domenico Solari, Ferdinando Caranci, Mario Cirillo, Lorenzo Ugga

**Affiliations:** 1 Department of Advanced Biomedical Sciences, University of Naples “Federico II”, Naples, Italy; 2 ASL NA3sud, U.O.S.M. 55-57 Torre del Greco, Ercolano, Italy; 3 Department of Medicine, Surgery and Dentistry, University of Salerno, Baronissi, Italy; 4 Department of Neuroradiology, University Hospital “San Giovanni Di Dio E Ruggi d’Aragona”, Salerno, Italy; 5 Department of Medical and Surgical Specialties, Radiological Sciences and Public Health, University of Brescia, Brescia, Italy; 6 Department of Neurosciences, Reproductive and Odontostomatological Sciences, University of Naples “Federico II”, Naples, Italy; 7 Department of Advanced Medical and Surgical Sciences, University of Campania “Luigi Vanvitelli”, P.zza L. Miraglia, 2, Naples, ZIP 80138 Italy

**Keywords:** Pituitary adenoma, Tumor consistency, Radiomics, Machine learning

## Abstract

**Purpose:**

Pituitary adenoma consistency significantly influences surgical strategy and outcomes, yet it cannot be reliably predicted using conventional imaging. By converting images into quantitative data, radiomics can identify imaging patterns that may not be distinguishable by standard visual interpretation. This study aims to evaluate the diagnostic performance of radiomics-based MRI models for predicting pituitary adenoma consistency, to determine its potential for presurgical planning.

**Materials and methods:**

A systematic search of PubMed, EMBASE, and Scopus was performed on 12/03/2025 following PRISMA-DTA guidelines and a pre-registered protocol (PROSPERO/CRD420251246028). Eligible studies applied radiomics and machine learning/deep learning to predict pituitary adenoma consistency. We performed random-effects meta-analyses to evaluate the area under the receiver operating characteristic (ROC) curve (AUC). A hierarchical summary ROC (HSROC) model estimated sensitivity and specificity. Risk of bias and study quality were assessed with QUADAS-2 and METRICS.

**Results:**

Fourteen studies were included. The pooled discriminative performance was good (AUC = 0.86,95% C.I. [0.76;0.92], *I*^2^ = 92.45%, *k* = 14). The HSROC model showed a sensitivity = 0.74,95% C.I. [0.56;0.87] and specificity = 0.79, 95% C.I. [0.73;0.84]. No significant performance differences were observed across algorithms, dimensionality, sequence, although T2w and combined sequence models showed higher AUC trends. Risk of bias was low to moderate, and study quality was good overall. Heterogeneity resulted high and small-study effect emerged in the main analysis.

**Conclusions:**

Radiomics-based MRI models demonstrate good diagnostic performance for predicting pituitary adenoma consistency and may support presurgical assessment and planning. However, substantial heterogeneity, evidence of small-study effects, and limited external validation warrant cautious interpretation of the pooled estimates and currently restrict clinical generalizability. Future studies should prioritize multicenter external validation and standardized radiomics workflows.

**Graphical abstract:**

**Figure:**
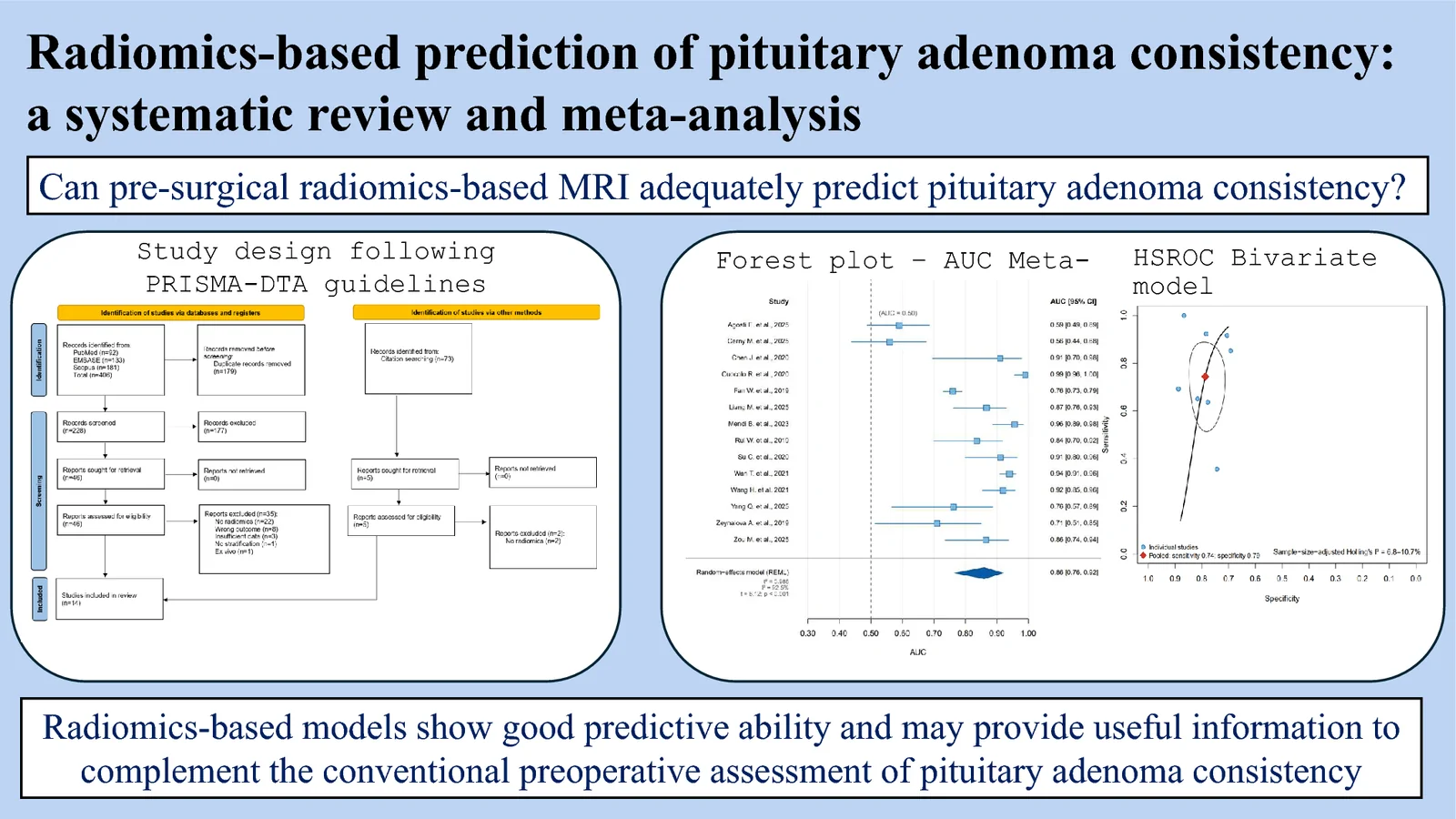

**Supplementary Information:**

The online version contains supplementary material available at https://doi.org/10.1007/s11547-026-02281-2.

## Introduction

Pituitary adenomas are the third most common type of brain tumor among adults [1]. Transsphenoidal surgery represents the preferred approach for the majority of lesions due to the location, since it provides the least disruptive access to the sella turcica, where the pituitary gland is located [2]. The surgical planning and operative strategy are influenced by tumor size and invasiveness, which can be easily assessed with pre-surgical imaging (e.g., Knosp grade to assess cavernous sinus invasion) and associate with the extent of resection [3]. Another factor that influences surgical planning and procedures is represented by the consistency of the lesion, since soft adenomas can be easily removed using standard transsphenoidal suction and curettage techniques, while fibrous ones are often hard to excise despite the use of dissectors and can require more aggressive dissection and extended surgical approaches [4, 5]. Furthermore, fibrous adenomas associate with lower gross resection rates and higher rates of post-surgical complications, including diabetes insipidus and cerebral nerve palsy, especially in tumors with cavernous sinus invasion [6, 7]. Fibrous adenomas represent about the 10% of all pituitary lesions [8], and their consistency cannot be reliably determined preoperatively using conventional magnetic resonance imaging (MRI) alone [7, 9]. Thereby, interest is growing regarding advanced image analysis, including radiomics and machine learning, to extract quantitative features that may help to pre-surgically predict the consistency of the adenomas. Radiomics is an imaging approach that transforms pixels and voxels in quantitative data that can capture variations in voxel intensity and spatial relationships that are often imperceptible to the human eye [10]. These characteristics may correlate with underlying tissue characteristics and can be used to develop predictive models for clinical features and outcomes, through machine learning and deep learning algorithms [11]. Preoperative prediction of adenoma consistency is crucial to improve surgical planning and patient outcomes. To date, however, the diagnostic performance of radiomics-based approaches for this purpose has not been systematically evaluated. The present study aims to systematically investigate the evidence about the predictive diagnostic performance of radiomics in pituitary adenoma consistency using a random-effects meta-analysis.

## Materials and methods

The present systematic review and meta-analysis was performed according to the 2020 edition of the Preferred Reporting Items for Systematic Reviews and Meta-Analyses, extension for Diagnostic Test Accuracy (PRISMA-DTA) guidelines [12], following a pre-registered protocol in PROSPERO (CRD420251246028). Amendments to the protocol are presented in Appendix S5.

### Literature search

We systematically searched PubMed, EMBASE, and Scopus until December 03rd, 2025. We supplemented a manual search screening the reference lists of included studies. No date or language restriction was applied. Appendix S1 provides full details on the search strategy selection process.

### Eligibility criteria

Inclusion criteria were: i) cross-sectional studies, (ii) reporting on individuals with pituitary adenomas pre-surgically evaluated with an MRI, (iii) reporting on radiomics, (iv) using machine learning (e.g., logistic regression, support vector machine, random forest classifier, LASSO regression, gradient boosting), deep learning, or quantitative MRI analysis to predict the consistency of the adenomas, (iv) reporting the area under the curve (AUC) of model and essential statistical data.

Exclusion criteria were: (i) reviews, (ii) case report/series, (iii) conference abstracts, (iv) ex vivo studies, (v) animal studies, (vi) not predicting consistency, (vii) without stratified data (if they report on multiple tumors), (viii) lack of adequate quantitative data.

### Study selection and data extraction

Two independent authors screened and retrieved titles and abstracts and performed the full-text screening. Disagreements were to be resolved by a third one. A standardized extraction form was used to collect the following data: author, year, country, study design, sample size, AUC, percentage of fibrous adenomas, predictive model, sequences employed (i.e., T1 weighted/T1w, T1w contrast enhanced/CET1w, T2 weighted/T2w), accuracy, dimensionality (i.e., 2D, 3D), true/false positives and negatives. When standard errors (SEs) of AUC estimates were not directly reported in the study, they were derived from 95% confidence intervals (95% C.I.) assuming a normal (i.e., Wald-type) approximation, unless otherwise specified. When 95% C.I.s were not available, the SEs were computed through the Hanley–McNeil method to analytically estimate the variance based on the observed AUC and the number of positive and negative cases [13].

### Outcomes

The primary outcome was the pooled AUC of the predictive models. We performed subgroup analyses to identify different machine learning algorithms, MRI sequence, dimensionality. As secondary outcome, an exploratory diagnostic accuracy analysis was conducted using a hierarchical summary receiver operating characteristic (HSROC) model [14].

### Risk of bias and study quality

Risk of bias was assessed using the Quality Assessment of Diagnostic Accuracy Studies-2 (QUADAS-2) tool [15]. In addition, the quality of radiomics reporting and methodological rigor was evaluated using the *MEthodological quality and Transparency in Radiomics Studies* (METRICS) tool [16, 17]. The distribution of METRICS scores was evaluated through the Shapiro–Wilk test, and the group differences were evaluated with a Student’s t test, considering the mean difference (MD), with the corresponding 95% C.I.

### Data analysis

A random-effects meta-analysis of AUC estimates was conducted to summarize the diagnostic performance across studies. The random-effects model assumes that observed estimates may vary across studies due to differences in patient populations, MRI acquisition parameters, segmentation strategies, feature extraction pipelines, model development approaches, and validation methods and accounts for the aforementioned variability [18]. The main effect size was the pooled AUC regarding the predicting performance in differentiating soft vs. fibrous adenomas. The main analysis accounted for a predefined hierarchy was applied to select one model from each study as follows: non-exploratory external validation, independent held-out test set, nested cross-validation, other internal cross-validation or resampling methods, and full-cohort analysis without independent validation, in descending order of priority. Within the highest available tier, the best-performing model identified by the original authors was selected. Heterogeneity was assessed with *I*^2^ and *τ*^2^ statistics. Publication bias was evaluated using funnel plots and Egger’s test for the primary outcome. Since logit transformation produces extreme values when AUCs approach 1.00, studies with confidence intervals reaching 1.00 may disproportionately influence funnel plot asymmetry and Egger’s test. Therefore, publication bias analyses were repeated after excluding these studies as a sensitivity analysis. We performed the following subgroup analyses for the primary outcome: (i) predictive algorithms, (ii) MRI sequences, (iii) dimensionality and a meta-regression regarding the percentage of fibrous adenomas in the sample. Statistical significance threshold was set at *p* = 0.05 for all analyses. Statistical analyses were performed using R 4.2.3 in R studio with the *metafor* 4.6–0 [19] and *mada* 0.5.12 [20] packages.

## Results

**Fig. 1:**
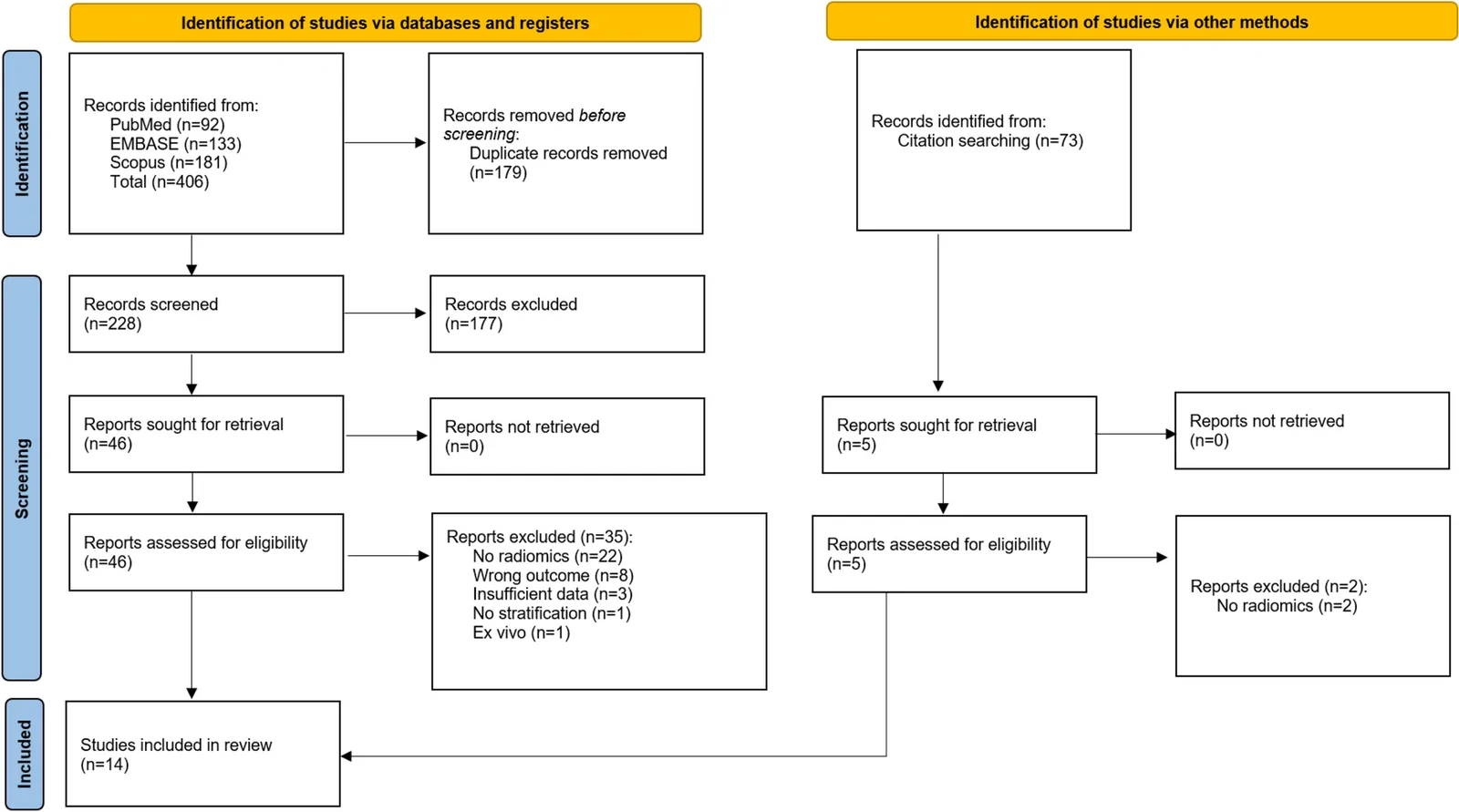
PRISMA 2020 flow diagram for new systematic reviews which included searches of databases, registers, and other sources. The flow diagram details the literature search and study selection process in the systematic review, with detail for each step

We identified 406 records from various sources, and after semi-automatic duplicate removal, we screened 228 records at a title abstract level. We sought 46 full texts for retrieval and assessed them for eligibility. Eventually, we found 14 eligible studies. Further information can be found in Fig. 1 (PRISMA flowchart).

**Fig. 2:**
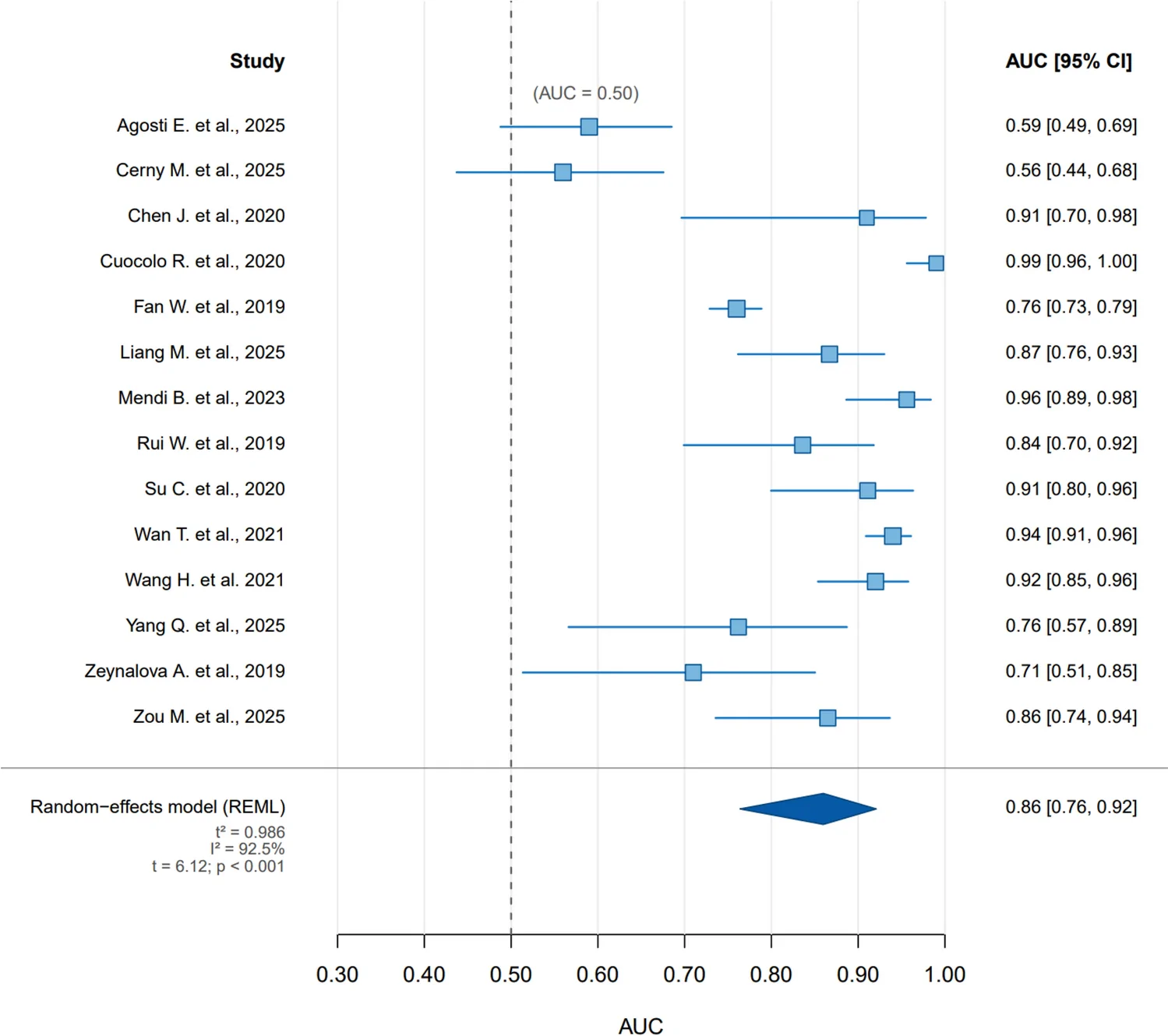
Forest plot, pooled area under the curve (AUC), main analysis. Each square represents the AUC estimate from individual studies, with horizontal lines showing the corresponding 95% C.I.s. The vertical dashed line marks the random chance level (i.e., AUC = 0.50). The diamond in the lower portion of the graph represents the pooled AUC. Its width indicates the 95% C.I

Characteristics of included studies are detailed in Table 1, and e-Tables 1, 2 excluded with reason at full-text screening are described in Appendix S5. The overall pooled discriminatory performance of the models predicting pituitary adenoma consistency was AUC = 0.86, SE = 0.03, 95% C.I. [0.76; 0.92], *I*^2 = ^92.45%, *τ*^2 = ^0.98, *k* = 14, which was significantly higher than random chance (*z* = 6.12, *p *< 0.01) (Fig. 2).

**Table 1:** Characteristics of included studies

Study	Country	Data source	Age range (Mean age ± SD)	% fibrous	Sample size	MRI	Sequence(s)	Model(s)	Validation	Accuracy	METRICS (%)
Cuocolo et al. 2020 [28]	Italy	Single institution	16-80y (52.17 + 14.64)	23.59	89 (57.3)	1.5–3 T	T2w	ET	Internal holdout (80:20); 5-fold CV for tuning	0.93	71.9
Zeynalova et al. 2019 [29]	Turkey	Single institution	NI (50.1 + NI)	24	55 (56.36)	1.5 T	T2	Deep learning	Nested internal CV (10-fold inner/10-fold outer)	0.725	66.7
Zou et al. 2025 [30]	China	Multicenter	NI (52.08 + 11.36)	26.3	133 (46.6) + 40 external	1.5–3 T	CET1w, T2w	SVM	External validation (*n* = 40); development cohort 7:3	0.838	93.1
Wan et al. 2021 [31]	China	Multicenter	NI	23.6	84 (NI)	3 T	T1w, T2w, CET1w	RF, SVM	Internal 60:10:30 split; 10-fold CV; 100 bootstraps	0.9	77.1
Cerny et al. 2025 [32]	Czech Republic	Multicenter	NI (53.5 ± 5.2)	22.5	542 (49)	NI	T2w, CET1w	RF	Internal holdout (70:30)	0.816	68.6
Rui et al. 2019 [33]	China	Single institution	29–69 (53 ± NI)	51	53 (58.5)	3 T	CE 3D-SPACE	LR	NI	0.77	52.5
Fan et al. 2019 [34]	China	Multicenter	2–48 (37 ± NI)	36.7	158 (60.13) + 30 external	3 T	T1w, T2w, CET1w	SVM	Internal holdout (100/58); external fusion model only (*n* = 30)	0.76	93.8
Chen et al. 2020 [35]	China	Single institution	21–78 (52 ± NI)	42.6	101 (48.5)	3 T	T1w, T2w, CET1w	LASSO	Internal holdout (72/29); 10-fold CV for tuning	NI	84.1
Wang et al. 2021 [36]	China	Single institution	NI (38.79 + 11.66)	34.7	170 (37.05)	3 T	T1w, T2w, CET1w	SVM, RF, ET, KNN, DT, GB, AB, MLP	Internal 10-fold CV; no independent test set	0.77	76.9
Agosti et al. 2025 [37]	Italy	Multicenter	>18y (NI)	25.9	174 (NI)	1.5–3 T	T1w, T2w, CET1w	ET	Internal holdout (60:40); 5-fold CV for tuning	0.63	78.8
Mendi et al. 2023 [38]	Turkey	Single institution	18–78(48.6 ± 13.7)	38.5	52 (52.7)	3 T	T2w	SVM, RF	ROI level 70:30 split; 10-fold bootstrap; patient separation NI	0.85	67.3
Liang et al. 2025 [39]	China	Dual center	>18y (51.9 + 12.2)	25.7	350 (50.3)	1.5–3 T	T1w, T2w, CET1w	LASSO, RF	Internal pooled dual-center holdout (7:3; 244/106)	0.88	84.1
Su et al. 2020 [40]	China	Single institution	24-80y (49.8 + 12.8)	26	50 (56)	3 T	T1w, T2w, CET1w	LR	NI	NI	58.0
Yang et al. 2025 [41]	China	Single institution	NI (45.9 + 13.9)	36.73	137 (43.1) + 10 external	1.5–3 T	T1w, T2w, CET1w	LASSO	External validation (*n* = 10); internal 108/29	0.72	95.4

AB = Adaptive Boosting; DT = Decision Tree Classifier; ET = Extra Tree Classifier; GB = Gradient Boosting; KNN = K-Nearest Neighbors; LASSO = Least Absolute Shrinkage And Selection Operator; LR = Logistic Regression; METRICS = METhodological *RadiomICs* Score; MLP = Multilayer Perceptron; MRI = Magnetic Resonance Imaging; NI = No information; RF = Random Forest; SD = Standard Deviation; SVM = Support Vector Machine

The meta-regression regarding the percentage of positive cases (i.e., with a fibrous adenoma) did not return a significant explanation of the variance (*p* = 0.79) (e-Fig. 1). A significant small-study effect emerged from the visual inspection of the funnel plot and Egger’s test results (*p* = 0.02) (e-Fig. 2a) which was not confirmed by the sensitivity excluding studies touching AUC=1.00 (*p* = 0.25) (e-Fig. 2b). The subgroup analysis considering the different predictive models did not show significant differences among the different algorithms (Q test *p* = 0.96) (e-Fig. 3). Regarding different sequences, although nonsignificant (Q test *p* = 0.66), a higher discriminative performance trend was highlighted by the subgroup analysis, where the combined model showed the highest performance (AUC = 0.85, 95% C.I. [0.78; 0.91], *k* = 11), followed by the T2w (AUC = 0.84, 95% C.I. [0.72; 0.92], *k* = 9). The T1w (AUC = 0.75, 95% C.I. [0.67; 0.82], *k* = 4) and CET1w (AUC = 0.77, 95% C.I. [0.72; 0.81], *k* = 5) sequences showed a lower performance (e-Fig. 4). Regarding dimensionality, the 2D vs. 3D analysis did not show significant differences (*p* = 0.99) (e-Fig. 5). The leave-one-out analysis failed to show single studies that significantly changed the pooled estimates (e-Fig. 6). The sensitivity analysis excluding the outliers (i.e., Agosti et al., Cerný et al., Cuocolo et al., and Mendi et al.) did not show significant differences about the pooled estimates, while heterogeneity decreased from *I*^2^ = 92.5 to 78.3%, although remaining substantial (e-Fig. 7).

**Fig. 3:**
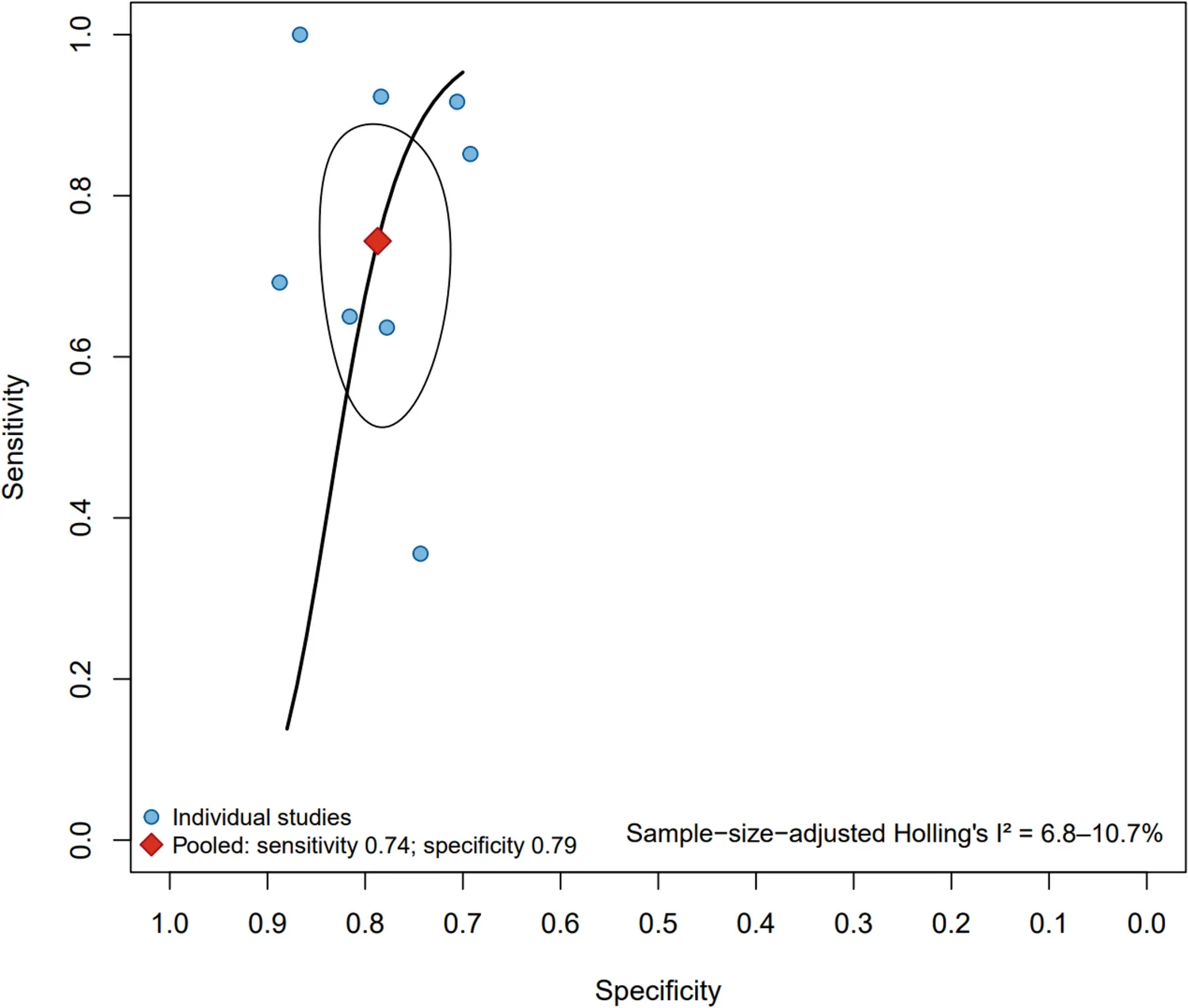
Hierarchical summary receiver operating characteristic (HSROC) bivariate random-effects model. Each gray point represents the sensitivity and specificity from an individual study. The solid curve shows the estimated HSROC function, and the ellipse represents the 95% confidence region around the summary operating point (i.e., red diamond), the pooled average diagnostic performance across studies

**Fig. 4:**
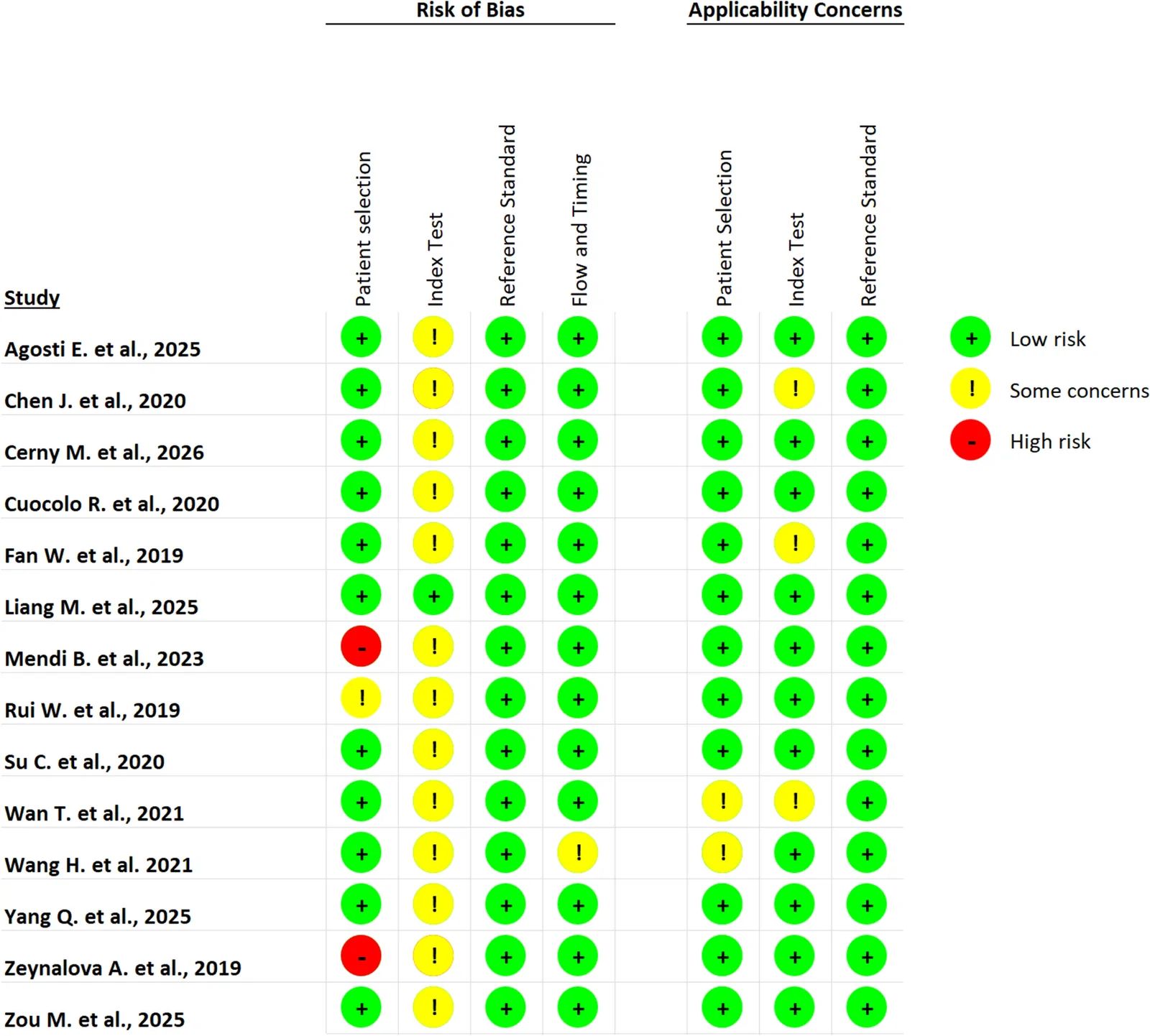
Quality Assessment of Diagnostic Accuracy Studies-2 (QUADAS-2). Green indicates low risk, yellow indicates some concerns, and red indicates high risk for each of the four risk of bias and three applicability concern domains

The bivariate HSROC model (logLik = 13.18, AIC = − 16.37, BIC = − 12.51, *k* = 8) showed a pooled mean sensitivity of 0.74, 95% C.I. [0.56; 0.87] and a pooled mean specificity of 0.79, 95% C.I. [0.73; 0.84] with a sample size-adjusted Holling estimate of heterogeneity *I*^2^ = 6.8–10.7% (Fig. 3).

### Risk of bias and study quality

Across studies, risk of bias and applicability were mainly low and moderate/unclear risks, with low percentage of high-risk studies, regarding the patient selection risk of bias domain. Applicability concerns were generally low, with no high-risk judgments observed (Fig. 4 and e-Fig. 8).

Across all included studies, the mean METRICS score was 76.0 ± 13.0, indicating good overall methodological quality (e-Table 3), with a normal distribution (*W* = 0.96, *p* = 0.72) (e-Fig. 9). METRICS scores showed an increasing trend over time (e-Fig. 10). Studies published after the introduction of the METRICS framework by Kocak et al., 2024 [16] showed higher scores than earlier studies (84.00 ± 10.90 vs. 71.53 ± 12.30), although this difference did not reach statistical significance (MD = 12.47, 95% CI [− 1.93; 26.86]; df = 12; *p* = 0.08) (e-Fig. 11). The overall quality did not significantly explain the heterogeneity in main model’s results (e-Fig. 12).

## Discussion

To the best of our knowledge, the current paper is the first meta-analysis to assess the available evidence on the diagnostic performance of radiomics-based approaches for the preoperative prediction of pituitary adenoma consistency. The pooled results indicate that radiomics and machine learning models show good overall discriminative performance, with a pooled AUC of 0.86, significantly higher than random classification (i.e., AUC = 0.50). The pooled sensitivity of 0.74 and specificity of 0.79 observed in the HSROC model indicate that radiomics-based models are able to identify fibrous adenomas with reasonable accuracy while maintaining adequate specificity. Clinically speaking, this result is particularly relevant, as false-negative predictions may lead to an underestimation of surgical complexity. Radiomics-based prediction could complement conventional MRI by providing additional information on tumor consistency before surgery, that is a relevant information that may help anticipate surgical complexity alongside size, extension, and cavernous sinus invasion [21]. In particular, identifying fibrous adenomas may assist surgeons in anticipating more difficult tumor debulking and a potentially lower likelihood of complete resection. The observed operating point suggests that radiomics may offer incremental value for risk stratification and surgical planning, rather than serving as a standalone determinant of surgical approach. The results of our study show that quantitative MRI-based approaches may provide clinically relevant information that may complement conventional imaging assessment. However, it must be remarked that, despite the favorable diagnostic performance, substantial heterogeneity was observed potentially reflecting the between-study variability, including differences in acquisition protocols, segmentation, feature extraction and selection, model development, and validation procedures. Furthermore, radiomics is a relatively young and rapidly evolving field, and heterogeneity in reporting quality persists, as evidenced by the radiomics-specific quality assessments, although improving over time. Our findings showed that the formalization of quality and transparency standards in radiomics (i.e., [16]) has contributed to the increase in overall study quality, providing a shared methodological reference for both the conduct of radiomics analyses and the reporting of their results. However, it must be remarked that the overall quality did not significantly explain heterogeneity in our sample, as the meta-regression failed to reach significance.

Subgroup analyses did not show significant differences among different algorithms, suggesting that model performance may be driven more by feature quality and pre-processing choices than by specific classifiers. This finding aligns with existing literature, where feature robustness, pre-processing, and validation rigor are seen as primary to the overall performance [22]. No significant differences emerged between 2 and 3D approaches, although this result should be interpreted cautiously given the limited number of studies in 3D subgroup, resulting in low statistical power. Regarding different MRI sequences, a trend toward higher discriminative performance was observed for combined models, followed by T2w imaging, although failing to reach statistical significance. T2w signal intensity is primarily determined by the T2 relaxation time, which reflects how freely water protons can move [23]. It is influenced by the molecular environment, including interactions with the collagen and extracellular matrix, as proved in other situations [24]. Fibrous pituitary adenomas present a higher expression of transforming grow factor β (TGFβ) [25] which promotes collagen synthesis, especially type I [26]. Collagen highly interacts with water, potentially in different ways based on its type ratio [27]. Thereby, modifications in collagen expression and increased tissue density can determine signal heterogeneity in fibrous adenomas, captured by the radiomic approach, especially in T2w sequences.

Clinically speaking, radiomics-based prediction of pituitary adenoma consistency may be implemented as a tool supporting conventional imaging for pre-surgical planning. Along with tumor size, extension, and cavernous sinus invasion, radiomics may help anticipate technical difficulty due to consistency, providing useful information for the operative strategy. However, further prospective multicenter studies with standardized validation may be needed before widespread clinical implementation.

The stability of the pooled AUC in the leave-one-out and outlier sensitivity analyses suggests that the overall estimate was not driven by any single study or by the identified outliers. Nevertheless, heterogeneity remained substantial also in the aforementioned analyses, suggesting that it may reflect multiple methodological and clinical differences across studies, including variations in imaging protocols, segmentation approaches, radiomic pipelines, model development, and study populations.

### Limitations of the study

The visual inspection of funnel plots and Egger’s test indicated the presence of a small-study effect, which may reflect the selective reporting of higher-performing models, lack of external validation, or publication bias favoring positive results. Reporting quality varied, and not all studies adhered to established radiomics methodological standards, as reflected by METRICS assessments. Furthermore, the high heterogeneity underscores the need for caution when interpreting pooled estimates, especially regarding the AUC analysis, since the bivariate HSROC model showed low/moderate heterogeneity.

## Conclusions

This systematic review and meta-analysis demonstrate that radiomics-based approaches applied to preoperative MRI achieve good overall diagnostic performance for predicting pituitary adenoma consistency. Mean sensitivity and specificity observed in hierarchical diagnostic accuracy analyses suggest that radiomics may provide clinically meaningful information to support preoperative risk stratification and surgical planning. Overall, radiomics may represent a useful adjunct to conventional imaging for the preoperative assessment of pituitary adenoma consistency. However, substantial heterogeneity and limited external validation currently preclude their clinical implementation. Prospective multicenter validation using standardized radiomics workflows is required before these models can be reliably integrated into clinical practice.

## Supplementary Information

Below is the link to the electronic supplementary material.

Supplementary file1 (DOCX 1718 kb)
