# Supplementary material for: Radiomics-based prediction of pituitary adenoma consistency: a systematic review and meta-analysis
Source: Radiol Med. 2026 Sep 18;131(10):1841–51. doi: 10.1007/s11547-026-02281-2 (PMC13668906; doi:10.1007/s11547-026-02281-2)
Supplement: Supplementary file 1 [file 11547_2026_2281_MOESM1_ESM.docx]

# Section 1 (S1) - Description of the search strategy & supplementary information of included studies

## Search strategy

*All searches were performed on the 03^rd^ of December, 2025*

**PubMed – records found: 92**

**(((((((((((((((("machine learning"[Title/Abstract]) OR ("deep learning"[Title/Abstract])) OR ("artificial intelligence"[Title/Abstract])) OR ("radiomic"[Title/Abstract])) OR ("radiomics"[Title/Abstract])) OR ("support vector machine"[Title/Abstract])) OR ("regression"[Title/Abstract])) OR ("linear discriminant"[Title/Abstract])) OR ("random forest"[Title/Abstract])) OR ("gradient boosting"[Title/Abstract])) OR ("neural network"[Title/Abstract])) OR ("**predict**"[Title/Abstract]))** OR **("**prediction**"[Title/Abstract]))** OR **("**predicting**"[Title/Abstract]))** OR **("**predictive**"[Title/Abstract])) AND ((((((((((("texture"[Title/Abstract]) OR ("textural"[Title/Abstract])) OR ("texturing"[Title/Abstract])) OR ("texturally"[Title/Abstract])) OR ("texturization"[Title/Abstract])) OR ("texturize"[Title/Abstract])) OR ("texturized"[Title/Abstract])) OR ("firm"[Title/Abstract])) OR ("soft"[Title/Abstract])) OR ("fibrous"[Title/Abstract])) OR ("hard"[Title/Abstract]))) AND (((((((("pituitary adenoma"[Title/Abstract]) OR ("pituitary adenomas"[Title/Abstract])) OR ("pituitary neuroendocrine tumor"[Title/Abstract])) OR ("pituitary tumor"[Title/Abstract])) OR ("pituitary cancer"[Title/Abstract])) OR ("prolactinoma"[Title/Abstract])) OR ("pituitary microadenoma"[Title/Abstract])) OR ("pituitary macroadenoma"[Title/Abstract]))**

**EMBASE – records found: 133**

('machine learning':ti,ab,kw OR 'deep learning':ti,ab,kw OR 'artificial intelligence':ti,ab,kw OR 'radiomic':ti,ab,kw OR 'radiomics':ti,ab,kw OR 'support vector machine':ti,ab,kw OR 'regression':ti,ab,kw OR 'linear discriminant':ti,ab,kw OR 'random forest':ti,ab,kw OR 'gradient boosting':ti,ab,kw OR 'neural network':ti,ab,kw OR predict:ti,ab,kw OR prediction:ti,ab,kw OR predictive:ti,ab,kw OR predicting:ti,ab,kw) AND ('texture':ti,ab,kw OR 'textural':ti,ab,kw OR 'texturing':ti,ab,kw OR 'texturally':ti,ab,kw OR 'texturization':ti,ab,kw OR 'texturize':ti,ab,kw OR 'texturized':ti,ab,kw OR 'firm':ti,ab,kw OR 'soft':ti,ab,kw OR 'fibrous':ti,ab,kw OR 'hard':ti,ab,kw) AND ('pituitary adenoma':ti,ab,kw OR 'pituitary adenomas':ti,ab,kw OR 'pituitary neuroendocrine tumor':ti,ab,kw OR 'pituitary tumor':ti,ab,kw OR 'pituitary cancer':ti,ab,kw OR 'prolactinoma':ti,ab,kw OR 'pituitary microadenoma':ti,ab,kw OR 'pituitary macroadenoma':ti,ab,kw)

**Scopus – records found: 181**

( TITLE-ABS-KEY ( ( "machine learning" OR "deep learning" OR "artificial intelligence" OR radiomic OR radiomics OR "support vector machine" OR "regression" OR "linear discriminant" OR "random forest" OR "gradient boosting" OR "neural network" OR predict OR prediction OR predicting OR predictive ) ) AND TITLE-ABS-KEY ( ( texture OR textural OR texturing OR texturally OR texturization OR texturize OR texturized OR firm OR soft OR fibrous OR hard ) ) AND TITLE-ABS-KEY ( ( "pituitary adenoma" OR "pituitary adenomas" OR "pituitary neuroendocrine tumor" OR "pituitary tumor" OR "pituitary cancer" OR "prolactinoma" OR "pituitary microadenoma" OR "pituitary macroadenoma" ) ) )

## e-box 1. Hits fetched by different databases/registers.

| **Databases, trial registers, and other sources** | | **Citations** |
| --- | --- | --- |
| **Databases:** | |  |
| PubMed | | 92 |
| EMBASE | | 133 |
| Scopus | 181 | |
| **Total (databases)** | | **406** |
|  | |  |
| **Other sources:** |  | |
| Citation searching | 73 | |
|  | |  |
| **Total hits:** | | **479** |

**e-table 1. Reference standards used to classify pituitary adenoma consistency**

| **Study** | **Reference source** | **Operational definition** | **Assessment, blinding, and adjudication** |
| --- | --- | --- | --- |
| **Cuocolo et al., 2020 [1]** | Intraoperative surgical assessment | Soft: removable with conventional curettage and suction. Fibrous: resistant/difficult to remove and requiring more complex maneuvers such as extracapsular dissection. | Blinded double-check by two pituitary surgeons with >10 years’ experience; no disagreements reported. |
| **Zeynalova et al., 2019 [2]** | Composite surgical and histopathological assessment | Surgical: soft if removable by aspiration/curettage; otherwise hard. Histology: H&E and Masson trichrome collagen scoring; total score ≤4 soft and 5-6 hard. | Two neurosurgeons and two pathologists assessed blinded data. A third pathologist adjudicated pathology discrepancies; surgical-pathology disagreement was resolved by consensus. |
| **Zou et al., 2025 [3]** | Postoperative histopathological collagen quantification | Masson trichrome staining; blue extracellular-matrix area measured with ImageJ in five random ×200 fields. Non-fibrous <15% collagen; fibrous ≥15%. | Assessor number, blinding, and adjudication were not reported. |
| **Wan et al., 2021 [4]** | Not clearly reported | Patients were categorized as soft or firm, but the source and operational criteria for consistency classification were not described clearly. Postoperative pathology confirmed the tumor diagnosis. | Assessor number, blinding, and adjudication were not reported. |
| **Černý et al., 2025 [5]** | Immediate postoperative surgeon assessment | Soft: aspirated or easily removed with suction/curettes. Firm: required extracapsular dissection, piecemeal removal, or ultrasonic aspiration. For cystic tumors, only the solid component was assessed. | Recorded directly after surgery; blinding and adjudication were not reported. |
| **Rui et al., 2019 [6]** | Intraoperative surgical assessment | Soft: easily removed by aspiration/curettage. Hard: not removable by aspiration/curettage and requiring piecemeal resection with a microdissector or forceps. | All operations by one surgeon; two neurosurgeons reviewed surgical notes and videos immediately after surgery. Histology was evaluated separately by a blinded neuropathologist. |
| **Fan et al., 2019 [7]** | Postoperative neurosurgical assessment | Bahuleyan classification: soft if suctionable with an aspirator; firm if not suctionable. | High-quality surgical video reviewed by two experienced neurosurgeons; blinding/adjudication were not clearly reported. |
| **Chen et al., 2020 [8]** | Intraoperative surgical assessment | Soft: flowed spontaneously and was easily removed by suction/curette. Hard: not removable by suction/curette, with strong traction and sometimes requiring electrocoagulation or sharp piecemeal cutting. | Assessor number, blinding, and adjudication were not reported. |
| **Wang et al., 2021 [9]** | Operative assessment | Bahuleyan classification: soft if suctionable with an aspirator; firm if not suctionable. | Assessor number, blinding, and adjudication were not reported. |
| **Agosti et al., 2025 [10]** | Operative reports plus intraoperative video review | Soft: easily removed using suction/curettage. Fibrous: denser and requiring meticulous extracapsular dissection or instruments such as microdissectors. | Two pituitary neurosurgeons with >10 years’ experience independently reviewed videos; disagreements were resolved jointly with the primary surgeon. |
| **Mendi et al., 2023 [11]** | Surgical notes | Bahuleyan classification: soft if easily and totally resected by aspiration; firm if only partially resectable or requiring piecemeal resection for complete excision. | Assessor number, blinding, and adjudication were not reported. |
| **Liang et al., 2025 [12]** | Post-resection assessment documented in the surgical record | Soft: easily aspirated/curetted. Firm: required piecemeal resection with a microdissector or tumor forceps. | Primary and assistant neurosurgeons assessed consistency after resection; blinding/adjudication were not reported. |
| **Su et al., 2020 [13]** | Immediate postoperative surgical assessment | Soft: easily removable by aspiration/curettage. Hard: not removable by aspiration/curettage and requiring piecemeal resection with a microdissector or forceps. | Two neurosurgeons (37 and 10 years’ experience) assessed together. Masson trichrome collagen analysis was descriptive and not the classification reference. |
| **Yang et al., 2025 [14]** | Postoperative review and neurosurgeons’ medical records | Tumor classified as soft when the soft component predominated and hard when the hard component predominated; the whole tumor was treated as one consistency category. | Assessor number, blinding, and adjudication were not reported; collagen was not quantified. |

Abbreviations: H&E, hematoxylin and eosin; ImageJ, image-analysis software; NR, not reported.

**e-table 2. HSROC data and reasons for exclusion from the exploratory analysis**

| **Study** | **Included in HSROC** | **TP** | **FP** | **TN** | **FN** | **2×2 evidence source (or reason for exclusion)** |
| --- | --- | --- | --- | --- | --- | --- |
| Agosti et al., 2025 | **Yes** | 16 | 29 | 84 | 29 | Direct confusion matrix |
| Černý et al., 2025 | **No** | / | | | | No patient-level 2×2 in article |
| Chen et al., 2020 | **Yes** | 11 | 5 | 12 | 1 | Approximate reconstruction from rounded metrics |
| Cuocolo et al., 2020 | **Yes** | 13 | 2 | 13 | 0 | Direct confusion matrix |
| Fan et al., 2019 | **Yes** | 13 | 7 | 31 | 7 | Reconstructed from class totals and rates |
| Liang et al., 2025 | **Yes** | 18 | 9 | 71 | 8 | Reconstructed from class totals and rates |
| Mendi et al., 2023 | **No** | / | | | | ROI-level performance only |
| Rui et al., 2019 | **Yes** | 23 | 8 | 18 | 4 | Reconstructed from class totals and rates |
| Su et al., 2020 | **Yes** | 12 | 8 | 29 | 1 | Reconstructed and outcome-reoriented |
| Wan et al., 2021 | **No** | / | | | | No unique test-set 2×2 |
| Wang et al., 2021 | **No** | / | | | | Insufficient classification data |
| Yang et al., 2025 | **Yes** | 7 | 4 | 14 | 4 | Reconstructed from class totals and rates |
| Zeynalova et al., 2019 | **No** | / | | | | Sample-level matrix only |
| Zou et al., 2025 | **No** | / | | | | No 2×2 data |

A 2×2 matrix was accepted only when it represented independent patients and was directly reported or uniquely reconstructable from class totals and performance measures. The **positive class** was deemed as the firm/hard/fibrous adenomas. For studies that treated soft consistency as the positive class, the matrix was re-oriented for consistency.

Decision thresholds were not reported, except for Rui et al. that stated that the Youden index was used without giving the numeric cutoff.

# Section 2 (S2) - Analyses

**e-figure 1. Meta-regression by fibrous adenoma % of the sample**

**e-figure 2a. Small-study effect, main model. Funnel plot and Egger’s test.** The test showed a significant small-study effect.

**e-figure 2b. Small-study effect, main model, removing studies with C.I.s touching AUC=1. Funnel plot and Egger’s test.** Since AUC values are bounded between 0 and 1, estimates approaching the upper boundary can become disproportionately influential after logit transformation. In particular, the near-perfect estimates reported by Cuocolo et al. and Mendi et al., whose confidence intervals approached or reached 1.00, could exert excessive leverage on the funnel-plot asymmetry and Egger regression. Therefore, in addition to the primary analysis including all studies, we performed a sensitivity analysis excluding these two studies to assess the robustness of the small-study-effect findings to this boundary-related distortion. The test failed to confirm a significant small-study effect.

**e-figure 3. Predictive radiomic models – AUC, subgroup analysis by model**

**e-figure 4. Predictive radiomic models – AUC, subgroup analysis by MRI sequence**

**e-figure 5. Predictive radiomic models – AUC, subgroup analysis by dimensionality**

**e-figure 6. Leave-one-out analysis.** The leave-one-out analysis failed to show a clear single-study contribute to heterogeneity.

**e-figure 7. Sensitivity analysis without outliers**

# Section 3 (S3) - Risk of bias and study quality evaluation

**e-box 2.** **Quality Assessment of Diagnostic Accuracy Studies – 2 (QUADAS-2)**

**e-figure 8. Risk of bias and Applicability concerns (QUODAS-2) across studies**

**e-table 3. METhodological RadiomICs Score (METRICS Tool v1.0)** **[1]**

| **Study** | I1 | I2 | I3 | I4 | I5 | I6 | I7 | I8 | I9 | I10 | I11 | I12 | I13 | I14 | I15 | I16 | I17 | I18 | I19 | I20 | I21 | I22 | I23 | I24 | I25 | I26 | I27 | I28 | I29 | I30 | Score |
| --- | --- | --- | --- | --- | --- | --- | --- | --- | --- | --- | --- | --- | --- | --- | --- | --- | --- | --- | --- | --- | --- | --- | --- | --- | --- | --- | --- | --- | --- | --- | --- |
| Agosti E. et al., 2025 | N | Y | Y | Y | Y | Y | Y | Y | / | Y | Y | Y | Y | Y | Y | Y | / | Y | Y | Y | Y | N | N | N | N | Y | N | N | N | Y | 78.8% |
| Cerny M. et al., 2025 | N | Y | Y | N | Y | Y | Y | Y | N | Y | Y | Y | Y | Y | Y | N | / | Y | Y | Y | N | N | N | N | N | Y | N | Y | Y | Y | 68.6% |
| Chen J. et al., 2020 | N | Y | Y | N | Y | Y | Y | Y | / | Y | Y | Y | Y | Y | Y | Y | / | Y | Y | Y | Y | N | Y | Y | Y | Y | N | N | N | N | 79.6% |
| Cuocolo R. et al., 2020 | N | Y | Y | N | Y | Y | Y | Y | / | Y | Y | Y | Y | Y | Y | Y | / | Y | N | Y | Y | N | N | N | N | Y | N | N | Y | Y | 71.9% |
| Fan W. et al., 2019 | N | Y | Y | Y | Y | Y | Y | Y | / | Y | Y | Y | Y | Y | Y | Y | / | Y | Y | Y | Y | Y | Y | Y | Y | Y | Y | N | N | N | 93.8% |
| Liang M. et al., 2025 | N | Y | Y | Y | Y | Y | Y | Y | / | Y | Y | Y | Y | Y | Y | Y | / | Y | Y | Y | Y | N | Y | Y | Y | Y | N | N | N | N | 84.1% |
| Mendi B. et al., 2023 | N | Y | Y | N | Y | Y | N | Y | / | Y | Y | Y | Y | Y | Y | Y | / | Y | N | Y | Y | N | N | N | N | Y | N | N | N | N | 67.3% |
| Rui W. et al., 2019 | N | Y | Y | N | Y | Y | Y | Y | / | Y | Y | Y | Y | Y | N | N | / | N | N | Y | N | N | N | N | N | N | N | N | N | N | 52.5% |
| Su C. et al., 2020 | N | Y | Y | N | Y | Y | Y | Y | / | Y | Y | Y | Y | Y | N | N | / | N | N | Y | Y | N | Y | N | Y | N | N | N | N | N | 58.0% |
| Wan T. et al., 2021 | N | Y | Y | N | Y | Y | Y | Y | / | Y | Y | N | Y | N | Y | Y | / | Y | N | Y | Y | N | Y | Y | N | Y | Y | N | N | N | 77.1% |
| Wang H. et al. 2021 | N | Y | Y | N | Y | Y | Y | Y | Y | Y | Y | Y | Y | N | Y | Y | Y | Y | Y | Y | N | N | Y | Y | Y | Y | N | Y | N | N | 76.9% |
| Yang Q. et al., 2025 | N | Y | Y | Y | Y | Y | Y | Y | / | Y | Y | Y | Y | Y | Y | Y | / | Y | Y | Y | Y | Y | Y | Y | Y | Y | Y | Y | N | Y | 95.4% |
| Zeynalova A. et al., 2019 | N | Y | Y | N | Y | Y | Y | Y | / | Y | Y | Y | Y | Y | Y | Y | / | N | N | Y | N | N | Y | Y | N | Y | N | N | N | Y | 66.7% |
| Zou M. et al., 2025 | N | Y | Y | Y | Y | Y | Y | Y | / | Y | Y | Y | Y | Y | Y | Y | / | Y | Y | Y | Y | Y | Y | N | Y | Y | Y | Y | Y | Y | 93.1% |

**Item explanation and scoring is available on official site (https://metricsscore.github.io/metrics/METRICS.html)**

**e-figure 9. Distribution of METRICS score (density).** The METRICS score follows a normal distribution.

**e-figure 10. Distribution of METRICS score per year**

**e-figure 11. METRICS score differences before and after** **METhodological RadiomICs standardization, Kocak B. et al., 2024** **[1]**

**e-figure 12. Main model meta-regression by METRICS.** The model failed to show statistical significance.

# Section 4 (S4) - Excluded with reason(s)

| **Excluded** | **Reason** |
| --- | --- |
| Cohen-Cohen S. et al., 2022 [2] | No AUC |
| Yamamoto J. et al. 2014 [3] | No radiomics |
| Alashwal M. et al., 2017 [4] | No radiomics |
| Yun et al. 2020 [5] | No radiomics |
| Romano A. et al., 2017 [6] | No radiomics |
| Pierallini et al., 2006 [7] | No radiomics |
| Acitores Cancela A. et al., 2024 [8] | No radiomics |
| Adamu M. et al., 2024 [9] | Wrong outcome |
| Baban M. et al., 2024 [10] | No radiomics |
| Barbosa M. et al., 2024 [11] | No radiomics |
| Cao Y. et al., 2025 [12] | Wrong outcome |
| Cappelletti M. et al., 2018 [13] | No radiomics |
| Cohen-Cohen S. et al., 2020 [14] | No radiomics |
| Conceição T. et al., 2025 [15] | No radiomics |
| Dheepak G. et al., 2024 [16] | Wrong outcome |
| Ding W. et al., 2021 [17] | No radiomics |
| Oh M. et al., 2011 [18] | No radiomics |
| Hassan R. et al., 2024 [19] | No radiomics |
| Jiang C. et al., 2023 [20] | Wrong outcome |
| Kamimura K. et al., 2021 [21] | No radiomics |
| Li P. et al., 2021 [22] | No radiomics |
| Liu C. et al., 2021 [23] | Wrong outcome |
| Malkauthekar M. et al., 2025 [24] | Wrong outcome |
| Micko A. et al., 2021 [25] | Ex vivo |
| Nie D. et al., 2022 [26] | No radiomics |
| Pereira F. et al., 2025 [27] | No radiomics |
| Smith K. et al., 2015 [28] | Insufficient data |
| Speckter H. et al., 2025 [29] | Wrong outcome |
| Su C. et al., 2023 [30] | No radiomics |
| Taghvaei M. et al., 2017 [31] | No radiomics |
| Wang X. Et al., 2023 [32] | Wrong outcome |
| Xu Q et al., 2022 [33] | No stratification |
| Yan P. et al., 2016 [34] | No radiomics |
| Yildrim M. et al., 2024 [35] | No radiomics |
| Yiping L., et al., 2016 [36] | No radiomics |
| Yu Y. Et al., 2025 [37] | No radiomics |
| Zhu H. et al., 2020 [38] | Insufficient data |

# Section 5 (S5) – Amendments to the protocol (CRD420251246028)

We performed, as a secondary outcome, a *hierarchical summary receiver operating characteristic (HSROC) model* to complement the primary AUC-based meta-analysis, as a pooling of sensitivity and specificity. We also performed a subgroup analysis considering dimensionality (i.e., 2D vs. 3D) and performed a meta-regression considering fibrous adenoma percentage of the sample for the primary outcome.

Following the Reviewers’ suggestions, we applied a predefined hierarchy when selecting the model for the primary analysis, choosing the best-performing eligible model within the highest available validation tier: non-exploratory external validation 🡪 held-out internal validation 🡪 cross-validation or other resampling methods 🡪 full-cohort analysis without independent validation.

We also performed sensitivity analyses to investigate potential sources of heterogeneity, including a leave-one-out analysis and an analysis excluding outliers, defined as studies whose individual 95% confidence intervals did not overlap the 95% confidence interval of the pooled estimate.

Since AUC values are bounded between 0 and 1, estimates approaching the upper boundary can become disproportionately influential after logit transformation. In particular, the near-perfect estimates reported by Cuocolo et al. and Mendi et al., whose confidence intervals approached or reached 1.00, could exert excessive leverage on the funnel-plot asymmetry and Egger regression. Therefore, in addition to the primary analysis including all studies, we performed a sensitivity analysis excluding these two studies to assess the robustness of the small-study-effect findings to this boundary-related distortion.

# Section 6 (S6) – References

1.	Kocak B, Akinci D’Antonoli T, Mercaldo N, Alberich-Bayarri A, Baessler B, Ambrosini I, et al. METhodological RadiomICs Score (METRICS): a quality scoring tool for radiomics research endorsed by EuSoMII. Insights into imaging. 2024;15(1):8.

2.	Cohen-Cohen S, Helal A, Yin Z, Ball MK, Ehman RL, Van Gompel JJ, et al. Predicting pituitary adenoma consistency with preoperative magnetic resonance elastography. J Neurosurg. 2022;136(5):1356-63.

3.	Yamamoto J, Kakeda S, Shimajiri S, Takahashi M, Watanabe K, Kai Y, et al. Tumor consistency of pituitary macroadenomas: predictive analysis on the basis of imaging features with contrast-enhanced 3D FIESTA at 3T. AJNR Am J Neuroradiol. 2014;35(2):297-303.

4.	Alashwah MA, Moharram MA, Allakany AS. Role of diffusion weighted magnetic resonance imaging DW-MRI in choice of the surgical approach for pituitary macroadenoma resection. Egyptian Journal of Radiology and Nuclear Medicine. 2017;48(4):983-9.

5.	Yun JJ, Johans SJ, Shepherd DJ, Martin B, Joyce C, Borys E, et al. The Utility of Using Preoperative MRI as a Predictor for Intraoperative Pituitary Adenoma Consistency and Surgical Resection Technique. J Neurol Surg B Skull Base. 2020;81(6):651-8.

6.	Romano A, Coppola V, Lombardi M, Lavorato L, Di Stefano D, Caroli E, et al. Predictive role of dynamic contrast enhanced T1-weighted MR sequences in pre-surgical evaluation of macroadenomas consistency. Pituitary. 2017;20(2):201-9.

7.	Pierallini A, Caramia F, Falcone C, Tinelli E, Paonessa A, Ciddio AB, et al. Pituitary macroadenomas: preoperative evaluation of consistency with diffusion-weighted MR imaging—initial experience. Radiology. 2006;239(1):223-31.

8.	Acitores Cancela A, Rodríguez Berrocal V, Pian Arias H, Díez Gómez JJ, Iglesias Lozano P. Development and validation of a prediction model for consistency of pituitary adenoma: the PiTCon score. Acta Neurochir (Wien). 2024;166(1):84.

9.	Adamu MJ, Kawuwa HB, Qiang L, Nyatega CO, Younis A, Fahad M, et al. Efficient and Accurate Brain Tumor Classification Using Hybrid MobileNetV2-Support Vector Machine for Magnetic Resonance Imaging Diagnostics in Neoplasms. Brain Sci. 2024;14(12).

10.	Baban MIA, Omer SN, AlZuhairy AKA, Mahmoud AA. The role of preoperative MRI in endoscopic transnasal transsphenoidal hypophysectomy of pituitary adenoma. Eur Arch Otorhinolaryngol. 2024;281(4):1961-9.

11.	Barbosa MA, Pereira EGR, da Mata Pereira PJ, Guasti AA, Andreiuolo F, Chimelli L, et al. Diffusion-weighted imaging does not seem to be a predictor of consistency in pituitary adenomas. Pituitary. 2024;27(2):187-96.

12.	Cao Y, Liang F, Zhao T, Han J, Wang Y, Wu H, et al. Brain tumor intelligent diagnosis based on Auto-Encoder and U-Net feature extraction. PLoS One. 2025;20(3):e0315631.

13.	Cappelletti M, Ruggeri AG, Spizzichino L, D'Amico A, D'Avella E, Delfini R. Fibrous Pituitary Macroadenomas: Predictive Role of Preoperative Radiologic Investigations for Proper Surgical Planning in a Cohort of 66 Patients. World Neurosurg. 2019;121:e449-e57.

14.	Cohen-Cohen S, Hughes JD, Yin Z, Ball MK, Huston J, Van Gompel JJ. Predicting pituitary adenomas consistency with preoperative magnetic resonance elastography. Journal of Neurological Surgery, Part B Skull Base. 2020;81.

15.	Conceição T, Silva J, Ruffini ML, Souza AM, Reis F, Antunes Á CM, et al. Testing magnetic resonance imaging parameters to predict pituitary macroadenoma consistency. Arq Neuropsiquiatr. 2025;83(11):1-7.

16.	Dheepak G, J AC, Vaishali D. Brain tumor classification: a novel approach integrating GLCM, LBP and composite features. Front Oncol. 2023;13:1248452.

17.	Ding W, Huang Z, Zhou G, Li L, Zhang M, Li Z. Diffusion-weighted imaging for predicting tumor consistency and extent of resection in patients with pituitary adenoma. Neurosurg Rev. 2021;44(5):2933-41.

18.	Enokizono M, Morikawa M, Ideguchi R, Uetani M, Matsuo T, Nagata I, et al. Preoperative evaluation of consistency of pituitary adenomas with T2 map and PROPELLER DWI. Neuroradiology Journal. 2010;23:390.

19.	Hassan RMA, Almalki YE, Basha MAA, Alduraibi SK, Hassan AH, Aboualkheir M, et al. Predicting the consistency of pituitary macroadenomas: the utility of diffusion-weighted imaging and apparent diffusion coefficient measurements for surgical planning. Diagnostics. 2024;14(5):493.

20.	Jiang C, Zhang W, Wang H, Jiao Y, Fang Y, Feng F, et al. Machine Learning Approaches to Differentiate Sellar-Suprasellar Cystic Lesions on Magnetic Resonance Imaging. Bioengineering (Basel). 2023;10(11).

21.	Kamimura K, Nakajo M, Bohara M, Nagano D, Fukukura Y, Fujio S, et al. Consistency of Pituitary Adenoma: Prediction by Pharmacokinetic Dynamic Contrast-Enhanced MRI and Comparison with Histologic Collagen Content. Cancers (Basel). 2021;13(15).

22.	Li P, Zhang D, Ma S, Kang P, Zhang C, Mao B, et al. Consistency of pituitary adenomas: Amounts of collagen types I and III and the predictive value of T2WI MRI. Exp Ther Med. 2021;22(5):1255.

23.	Liu CX, Heng LJ, Han Y, Wang SZ, Yan LF, Yu Y, et al. Usefulness of the Texture Signatures Based on Multiparametric MRI in Predicting Growth Hormone Pituitary Adenoma Subtypes. Front Oncol. 2021;11:640375.

24.	Malakuthekar M, Gulve A, Deshmukh R. A novel Probabilistic Bi-Level Teaching–Learning-Based Optimization (P-BTLBO) algorithm for hybrid feature extraction and multi-class brain tumor classification using ResNet-50 and GLCM. Journal of Engineering and Applied Science. 2025;72(1).

25.	Micko A, Placzek F, Fonollà R, Winklehner M, Sentosa R, Krause A, et al. Diagnosis of Pituitary Adenoma Biopsies by Ultrahigh Resolution Optical Coherence Tomography Using Neuronal Networks. Front Endocrinol (Lausanne). 2021;12:730100.

26.	Nie D, Zhao P, Li C, Liu C, Zhu H, Gui S, et al. Application of "mosiac sign" on T2-WI in predicting the consistency of pituitary neuroendocrine tumors. Front Surg. 2022;9:922626.

27.	Pereira FV, Ferreira D, Garmes H, Zantut-Wittmann DE, Rogério F, Fabbro MD, et al. Machine Learning Prediction of Pituitary Macroadenoma Consistency: Utilizing Demographic Data and Brain MRI Parameters. J Imaging Inform Med. 2025.

28.	Smith KA, Leever JD, Chamoun RB. Prediction of Consistency of Pituitary Adenomas by Magnetic Resonance Imaging. J Neurol Surg B Skull Base. 2015;76(5):340-3.

29.	Speckter H, Radulovic M, Lazo E, Hernandez G, Bido J, Rivera D, et al. Prediction of Pituitary Adenoma's Volumetric Response to Gamma Knife Radiosurgery Using Machine Learning-Supported MRI Radiomics. J Clin Med. 2025;14(9).

30.	Su CQ, Wang BB, Tang WT, Tao C, Zhao P, Pan MH, et al. Diffusion-relaxation correlation spectrum imaging for predicting tumor consistency and gross total resection in patients with pituitary adenomas: a preliminary study. Eur Radiol. 2023;33(10):6993-7002.

31.	Taghvaei M, Tajik F, Zeinalizadeh M, Ardalan FA, Ghanaati H. Is there any congruity between tumor consistency in pituitary macroadenomas and preoperative diffusionweighted imaging and apparent diffusion coefficient (ADC) map? Archives of Neuroscience. 2017;4(4).

32.	Wang X, Dai Y, Lin H, Cheng J, Zhang Y, Cao M, et al. Shape and texture analyses based on conventional MRI for the preoperative prediction of the aggressiveness of pituitary adenomas. Eur Radiol. 2023;33(5):3312-21.

33.	Xu Q, Xu H, Liu J, Zhou M, Li M, Xu J, et al. MR Image Classification for Brain Tumor Texture Based on Pseudo-Label Learning and Optimized Feature Extraction. Computational and Mathematical Methods in Medicine. 2022;2022.

34.	Yan PF, Yan L, Zhang Z, Salim A, Wang L, Hu TT, et al. Accuracy of conventional MRI for preoperative diagnosis of intracranial tumors: A single center report of 762 cases. International Journal of Surgery. 2016;36:109-17.

35.	Yildirim MS, Schmidbauer VU, Micko A, Lechner L, Weber M, Furtner J, et al. Multi-Dynamic-Multi-Echo-based MRI for the Pre-Surgical Determination of Sellar Tumor Consistency: a Quantitative Approach for Predicting Lesion Resectability. Clin Neuroradiol. 2024;34(3):663-73.

36.	Yiping L, Ji X, Daoying G, Yin Y. Prediction of the consistency of pituitary adenoma: A comparative study on diffusion-weighted imaging and pathological results. Journal of Neuroradiology. 2016;43(3):186-94.

37.	Yu Y, Feng B, Ren Y, Yu P, Zhu Y. The utilization of preoperative DWI for pituitary macroadenoma surgical planning: Implications for neuroendoscopic surgery. Endocrine. 2025;90(2):844-51.

38.	Zhu H, Fang Q, Huang Y, Xu K. Semi-supervised method for image texture classification of pituitary tumors via CycleGAN and optimized feature extraction. BMC Med Inform Decis Mak. 2020;20(1):215.

# Section 7 (S7) – PRISMA-DTA checklist

**Section/topic**

| **Section/topic** | **#** | **PRISMA-DTA Checklist Item** | **Reported on page #** |
| --- | --- | --- | --- |
| **TITLE / ABSTRACT** | | |  |
| Title | 1 | Identify the report as a systematic review (+/- meta-analysis) of diagnostic test accuracy (DTA) studies. | 1 |
| Abstract | 2 | Abstract: See PRISMA-DTA for abstracts. | 2 |
| **INTRODUCTION** | | |  |
| Rationale | 3 | Describe the rationale for the review in the context of what is already known. | 3 |
| Clinical role of index test | D1 | State the scientific and clinical background, including the intended use and clinical role of the index test, and if applicable, the rationale for minimally acceptable test accuracy (or minimum difference in accuracy for comparative design). | 3 |
| Objectives | 4 | Provide an explicit statement of question(s) being addressed in terms of participants, index test(s), and target condition(s). | 3 |
| **METHODS** | | |  |
| Protocol and registration | 5 | Indicate if a review protocol exists, if and where it can be accessed (e.g., Web address), and, if available, provide registration information including registration number. | 4 |
| Eligibility criteria | 6 | Specify study characteristics (participants, setting, index test(s), reference standard(s), target condition(s), and study design) and report characteristics (e.g., years considered, language, publication status) used as criteria for eligibility, giving rationale. | 4 |
| Information sources | 7 | Describe all information sources (e.g., databases with dates of coverage, contact with study authors to identify additional studies) in the search and date last searched. | 4 |
| Search | 8 | Present full search strategies for all electronic databases and other sources searched, including any limits used, such that they could be repeated. | Appendix S1 |
| Study selection | 9 | State the process for selecting studies (i.e., screening, eligibility, included in systematic review, and, if applicable, included in the meta-analysis). | 4 & Fig. 1 |
| Data collection process | 10 | Describe method of data extraction from reports (e.g., piloted forms, independently, in duplicate) and any processes for obtaining and confirming data from investigators. | 4 |
| Definitions for data extraction | 11 | Provide definitions used in data extraction and classifications of target condition(s), index test(s), reference standard(s) and other characteristics (e.g. study design, clinical setting). | 4 |
| Risk of bias and applicability | 12 | Describe methods used for assessing risk of bias in individual studies and concerns regarding the applicability to the review question. | 5 |
| Diagnostic accuracy measures | 13 | State the principal diagnostic accuracy measure(s) reported (e.g. sensitivity, specificity) and state the unit of assessment (e.g. per-patient, per-lesion). | 4-5 |
| Synthesis of results | 14 | Describe methods of handling data, combining results of studies and describing variability between studies. This could include, but is not limited to: a) handling of multiple definitions of target condition. b) handling of multiple thresholds of test positivity, c) handling multiple index test readers, d) handling of indeterminate test results, e) grouping and comparing tests, f) handling of different reference standards | 5 |

| **Section/topic** | **#** | **PRISMA-DTA Checklist Item** | **Reported on page #** |
| --- | --- | --- | --- |
| Meta-analysis | D2 | Report the statistical methods used for meta-analyses, if performed. | 5 |
| Additional analyses | 16 | Describe methods of additional analyses (e.g., sensitivity or subgroup analyses, meta-regression), if done, indicating which were pre-specified. | 5 |
| **RESULTS** | | |  |
| Study selection | 17 | Provide numbers of studies screened, assessed for eligibility, included in the review (and included in meta-analysis, if applicable) with reasons for exclusions at each stage, ideally with a flow diagram. | 6 & Tab 1 |
| Study characteristics | 18 | For each included study provide citations and present key characteristics including: a) participant characteristics (presentation, prior testing), b) clinical setting, c) study design, d) target condition definition, e) index test, f) reference standard, g) sample size | Tab 1 |
| Risk of bias and applicability | 19 | Present evaluation of risk of bias and concerns regarding applicability for each study. | 6-7, Fig.4 & App S2 |
| Results of individual studies | 20 | For each analysis in each study (e.g. unique combination of index test, reference standard, and positivity threshold) report 2x2 data (TP, FP, FN, TN) with estimates of diagnostic accuracy and confidence intervals, ideally with a forest or receiver operator characteristic (ROC) plot. | Fig. 2-3, Table 1 |
| Synthesis of results | 21 | Describe test accuracy, including variability; if meta-analysis was done, include results and confidence intervals. | 6-7, Fig. 2-3 |
| Additional analysis | 23 | Give results of additional analyses, if done (e.g., sensitivity or subgroup analyses, meta-regression; analysis of index test: failure rates, proportion of inconclusive results, adverse events). | 6-7, Fig. 2-3, Suppl. |
| **DISCUSSION** | | |  |
| Summary of evidence | 24 | Summarize the main findings including the strength of evidence. | 8-9 |
| Limitations | 25 | Discuss limitations from included studies (e.g. risk of bias and concerns regarding applicability) and from the review process (e.g. incomplete retrieval of identified research). | 9 |
| Conclusions | 26 | Provide a general interpretation of the results in the context of other evidence. Discuss implications for future research and clinical practice (e.g. the intended use and clinical role of the index test). | 9 |
| **FUNDING** | | |  |
| Funding | 27 | For the systematic review, describe the sources of funding and other support and the role of the funders. | Decl. statement |

**#**
